# Nurses’ perspective of conducting family conversation

**DOI:** 10.3402/qhw.v11.30867

**Published:** 2016-04-20

**Authors:** Åsa Dorell, Ulrika Östlund, Karin Sundin

**Affiliations:** 1 Department of Nursing, Umeå University, Örnsköldsvik, Sweden; 2 Country Council Region Gävleborg, Gävle, Sweden

**Keywords:** Family nursing, family systems nursing, family health conversation, intervention, older people, relatives, residential home, support, qualitative content analysis

## Abstract

**Introduction:**

Nurses are in a prime position to manage to support families that have a family member living in a residential home for older people. Nurses’ attitudes about meeting patients’ family members vary. Studies describe that some nurses consider family members as a burden. But some nurses consider family members a resource and think it is important to establish good relationships with them.

**Aim:**

The aim of this study was to describe how registered nurses (RNs) experienced to participate in and conduct the intervention Family Health Conversations (FamHCs) with families in residential homes for older people.

**Methods:**

The intervention FamHC was accomplished at three residential homes for older people. FamHC is a family systems nursing (FSN) intervention developed to support families facing the ill health of someone in the family. One RN from each residential home conducted the conversations. The RNs wrote diary notes directly after each conversation. The RNs were also interviewed 1 month after they had each conducted four FamHCs. The diary notes and the interviews were analysed separately by qualitative content analysis, and the findings were then summarized in one theme and further discussed together.

**Findings:**

The main findings were that the RNs experience the conversations as a valuable professional tool involving the whole family. The RNs grasped that silence can be a valuable tool and had learned to attentively listen to what the families were saying without interrupting, allowing them and the families to reflect upon what the family members said.

**Conclusion:**

The findings show that the FamHC can be helpful for RNs in their work, helping them to perceive and understand the needs and desires of the families.

Having a family member moving into or living at a residential home for older people can be demanding both for the resident and for the entire family (Eika, Espnes, Söderhamn, & Hvalvik, 2014; Opara & Jaracz, 2010). The challenges for the family involve feelings of guilt and failure, as well as lack of control over the situation and an inability to influence the care of their sick family member (Bramble, Moyle, & McAllister, 2009; Høgsnes, Melin-Johansson, Norbergh, & Danielson, 2014). The registered nurse (RN) involved in the care of the old person is in an ideal position to support and empower families with a chronically sick family member (Moore & McQuestion, 2012). Studies have found that nurses consider it important to establish a good relationship with families in residential care for older persons. However, nurses sometimes find families challenging (Åstedt-Kurki, Paavilainen, Tammentie, & Paunonen-Ilmonen, 2001; Hertzberg, Ekman, & Axelsson, 2003). Nurses believe it is important to develop approaches that improve collaboration with families (e.g., LeGrow & Rossen, 2005; Weman, Kihlgren, & Fagerberg, 2004). The focus in residential nursing care, however, has traditionally been on the patient, with the family only as context.

Within a family systems nursing (FSN) approach, both the patient and other family members are viewed simultaneously and as a unit by the nurses. The relationship, collaboration, and communication within families and between families and nurses is essential within FSN (Wright & Leahey, 2013). A review study reported systemic responses after families participated in FSN, such as improved understanding and enhanced coping within families. Furthermore, families cared more about and for the family as a whole, as well as about and for each other. The families’ emotional well-being improved and so did their interactions within and outside the family (Östlund & Persson, 2014). Other studies have also shown a positive impact on families’ after their participation in FSN interventions in different contexts (e.g., Årestedt, Persson, & Benzein, 2014; Benzein, Olin, & Persson, 2015; Kamban & Svavarsdottir, 2013; Simpson, Yeung, Kwan, & Wah, 2006; Sundin et al., 2015; Sveinbjarnardottir, Svavarsdottir, & Wright, 2013). This could be significant for the residents, their families, and the RNs, as FSN has been shown to improve emotional well-being, family function, and improved family–nurse collaboration (Duhamel, Dupuis, Turcotte, Martinez, & Goudreau, 2015; Östlund & Persson, 2014). Although there is growing evidence of the value of FSN intervention for families, there is still a gap of knowledge regarding nurses’ experiences of FSN intervention in the context of residential care. Research about nurses’ attitudes regarding FSN has previously been performed in educational settings, in the context of pediatrics, psychiatry, intensive care, and surgery (Blöndal et al., 2014). Studies have shown that nurses have a positive attitude toward family involvement (Söderström, Benzein, & Saveman, 2003). Nurses report that they gain a better understanding of the family situation using FSN approaches (Braun & Foster, 2011; Leahey & Svavarsdottir, 2009).

One example of an FSN intervention is the family health conversation (FamHC) (Benzein, Hagberg, & Saveman, 2008; Östlund, Bäckstrom, Lindh, Sundin, & Saveman, 2015). The FamHC intervention consists of a series of three nurse-led 1-h conversations with the families at 2-week intervals. The conversations have a systemic approach (Maturana, 1988; Öqvist, 2008) to the families and thus with reference to all the significant family members. The focus is on the relationship within the family and highlights all family members’ beliefs (Wright & Bell, 2009) and experiences about having an ill family member. FamHC aims to create an awareness of own and other family members’ existing beliefs and a context for change in relation to the problems described by the family. FamHC is theoretically grounded in a slutogenetic approach (Antonovsky, 1987) to support and strengthen the resources, both inside and outside the families. The core components for conducting the family conversations begin with reflecting the family expectations for the conversations and exploring the family structure, such as jointly prioritizing which problem(s) most need to be discussed. All family members are given space within the conversations and have the opportunity to narrate their experiences. Each family member's view is acknowledged as equally valid. Commendations are given and the suffering is acknowledging (Östlund et al., 2015). Narrating and reflective thinking are emphasized (Andersen, 1995), and all family members are invited to reflect on each other's narratives (Östlund et al., 2015). By narrating, the family members could share each other's perspectives and begin to reflect, thus enabling them to find new alternatives or meanings. These new alternatives or meanings may mediate a better understanding of one's own and others’ perspectives (Ricæur, 1992) and are assumed to have a great impact on the healing process through family members finding new alternatives and identifying new associations. Thus, FamHC is supposed to sustain health, promote healing, and relieve the suffering of the family. Having good communication and support from the nurse is important for helping families feel involved (Lethin, Hallberg, Karlsson, & Janlov, 2015). Therefore, it is important for nurses to expand the focus for care in order to support families with an ill family member living in a residential home for older people. This supports the need for interventions to minimize health risks to both the individual family members and the family unit.

To the best of our knowledge, there are no existing studies about the perspectives of RNs on conducting conversations using an FSN approach with families in the context of residential homes for older people. To address this lack of research, the aim of the present study was to describe how RNs experienced to participate in and conduct the intervention FamHCs with families in residential homes for older people.

## Method

### Participants and setting

The intervention was performed at three residential homes for older people in northern Sweden. A total of three RNs were recruited to perform FamHCs—one RN from each of the three intervention units. The first three RNs who reported an interest in participating and met the inclusion criteria were selected. The inclusion criteria were that the RNs had worked 2 years or longer in municipal senior care. They were all women aged 40–51 years, and they had worked as RNs for 5–15 years. This study included a total of 12 families comprising 24 family members who participated in FamHCs. The recruitment of the family members was conducted by the heads of the residential homes, together with the participating nurses. The participating family members included 22 women (five wives and 17 daughters) and two men (sons) ranging from 39 to 84 years old (Md = 55). As shown in Table I, the related residents were between 74 and 89 years old.

**Table I:** Demographics of the participating families and residents.

Family	Sex, age, and diagnosis of the resident	Years lived in the residential home for older people	Family members’ relation
A	Male, 87, dementia	3 years	Wife
B	Female, 81, dementia	1 year	Daughter
C	Male, 81, dementia	2 years	Daughter, daughter
D	Male, 76, dementia, heart disease	1 year	Wife, daughter
E	Male, 86, stroke, dementia Female, 82, dementia	5 years 1 year	Daughter
F	Male, 84, dementia	3 years	Wife, daughter, son
G	Female, 81, stroke	1 year	Daughter
H	Male, 84, stroke	6 month	Wife, daughter
I	Male, 76, stroke	1 year	Wife, son
J	Male, 80, stroke	2 years	Wife, daughter, daughter
K	Male, 89, stroke	2 years	Wife, daughter, daughter, daughter
L	Male, 82, dementia	1 year	Daughter, daughter

### Intervention

Before the RNs conducted the FamHC interventions with the families, they underwent education and training in FamHC given by the members of the research group. The RNs participated in weekly educational activities over a 2-month period. The purpose of the education was skill development and knowledge of how to perform the FamHC series. This training had content that was similar to the regular education on FSN and FamHC delivered at the university and contained theories about system theory, communication theory, and reflection theory (Lindh et al., 2013). Furthermore, role playing was practiced for skill development.

The three participating RNs, in pairs of three nurses from the research group, conducted the FamHC intervention with four families from each unit. The intervention included a series of three 1-h conversations at 2-week intervals. Each of the three conversations had a different topic and was intended to focus on what the families considered was important to talk about. The first conversation invited each family member to tell his or her experiences in the current life situation and listen to the other family members’ stories. The second conversation began with an opportunity to reflect on the first conversation and then turned its focus to suffering, problems, and beliefs. The third conversation focused on family strengths and resources, and on the future (Benzein et al., 2008; Östlund et al., 2015). The conversation series with the families was concluded by sending a “closing letter” within 2 weeks of the date of the last conversation to all participating family members. In the closing letter, the nurses provide written reflections on what happened over the three conversations, highlighting the families’ resources and strengths, and acknowledging each family member's suffering (Bell, Moules, & Wright, 2009). Ethical approval was obtained from the Regional Ethical Review Board of the University (xxxx-xxx-xxx).

### Data collection

The data presented and analysed were drawn from both diary notes and interviews.

#### Diary notes

The RNs were asked to write diary notes (Dahlberg, 2008) about what they experienced directly after conducting each conversation. Meaningful data can be collected with diary notes that would be difficult for the participant to remember after time has elapsed (Thomas, 2015). This was to capture their spontaneous experiences of conducting the FamHC. In total, 36 diary notes, each about one-half to one page in length, were collected.

#### Interviews

Semi-structured interviews with open-ended questions were conducted individually with the RNs, 1 month after they completed FamHC series with four families each, to allow them to narrate freely about their experiences (Polit & Beck, 2012). The interview guide covered questions about the RNs’ experiences in conducting FamHC, possible changes in their relationships with the families, positive or negative opinions on experiences during the conversation, how they distinguished these conversations from other types of communication used in their work, and thoughts about how using FamHC influenced their work. The interviewer was a person external to the research group and had no relationship with the participating RNs. The interviews took place in a conversation room in the residential home, lasted about 60 min, and were rich in content.

### Data analysis

The diary notes and the semi-structured interviews were analysed separately using inductive content analysis to find the RNs’ experiences with conducting FamHC (Elo & Kyngäs, 2008). The analysis for the two datasets followed a similar process.

The diary notes were analysed from the original diary text. Each diary was read through several times to get an overall understanding of the content in all diaries together as a whole text. Then, the meaning units were identified based on the aim of the study. A meaning unit is a unit of sentences that contains elements related to each other through content (Graneheim & Lundman, 2004). The meaning units were condensed and coded, then grouped by content into subcategories, and further abstracted into categories. Then, the original diary text was reread to verify and refine the categories that emerged.

The interviews were digitally taped and transcribed verbatim. Interview transcripts were first read several times to obtain an overall understanding of the content. Then, the interview texts were divided into meaning units. The meaning units were condensed and coded and then grouped by content into subcategories, and further abstracted into categories. The categories were compared to the original text.

Finally, one overall theme (Baxter, 1994) emerged from findings from the two separate analysis, that is, when going through the categories developed from both the diary notes and the interviews.

## Findings

The findings are presented through one theme that emerged from the perspectives of the diary notes and the interviews, separately.

### Comprehensive theme

The nurses’ experiences in participating and conducting FamHCs could be metaphorically described in a theme as

Feeling content with family conversations as a professional tool.

### Findings from the diary notes

Diary notes from the RNs’ primary written experiences, as they appeared directly after each conversation, are presented in three categories with associated subcategories (see Table II).

**Table II:** Theme, categories, and subcategories emerged from the RNs’ diary notes.

Theme	Categories	Subcategories
Feeling content with family conversations as a professional tool	A new approach to conversations	From fear to curiosity Amazement over the conversation content
	Listening as an art	Concerns around what attentive listening brings to the surface Practice in listening generates progress
	The power of reflection	Reflection shows family needs Reflection as a new tool

### A new approach to conversation

#### From fear to curiosity

The RNs described the first session in the conversation series as something new, a conversation different from what they usually held with families. The RNs expressed that they felt tense and nervous before the first conversation. They expressed that they were not used to posing open and reflection-based questions that facilitate narration. Silence during the conversations was experienced as stressful and challenging in the beginning of the conversation series. They described a shift in their feelings after conducting a couple of conversations, from feeling strained to feeling contented while conducting FamHCs. As they became more experienced in conducting the conversations, the RNs noted that they were looking forward to the second conversation with the families because the first sessions had aroused a curiosity to know more about how the families perceived their situations.

When there was moment of silence, I felt stressed at first.

#### Amazement over the conversation content

The RNs stated that their opinions of the families changed during the conversations. The families showed a vulnerability that was new to the RNs. They noted that seeing that the family members had such a great need to talk and tell their story was a discovery.

I got to see a different side of the family members; they showed a vulnerability I hadn't seen before.

### Listening as an art

#### Concerns around what attentive listening brings to the surface

The families expressed a lot of feelings and emotional thoughts that caused apprehension among the RNs. The RNs expressed feelings of dread, as well as fears of not being able to take care of the family's needs.

Things came up during the conversation that left me thinking, ‘What we have done?’

#### Practice in listening generates progress

The RNs described that being a conversational leader and simultaneously listening to what the families said in order to create a whole picture of the situation was difficult at the beginning. It became easier after they had conducted a couple of conversations.

I was the conversation leader and it was difficult.

### The power of reflection

#### Reflection shows family needs

During the third sessions in the conversation series, the RNs were surprised to find that when they posed a reflection question, the families started to narrate things they had never talked about before. Toward the end of the conversation series, it became clear to the RNs that they had previously failed to see the needs of families and to talk about thoughts and problems with families.

I was surprised by how much can come up from a question asked in the right way.

#### Reflection as a new tool

The RNs could see that the concerns they had in previous conversations over the fear and distress about the emotions unraveled by conversations had shifted, so that the reflections made in the conversations came to be seen as a tool for the RNs in their work with families. The FamHCs gave the RNs an opportunity to help families reflect upon and change their situations, and thereby improve family health.

I think the conversation has given a lot. I feel that a whole new background to the family members has been added into our relationship.

### Findings from interviews

The RNs’ reflective experiences as they appeared in interviews 1 month after conducting four FamHC series are presented in three categories with associated subcategories (see Table III).

**Table III:** Theme, categories and subcategories emerged from the interviews.

Theme	Categories	Subcategories
Feeling content with family conversations as a professional tool	A different conversation	Surprise over the families’ approach in the conversation Silence as a tool Focus on the family
	Conversation as a creator of relationship	Improved understanding of the families Family as a resource
	To grow in the role of nurse	Improved self-confidence when conducting the FamHC Developing communication skills Being mentored Apply their skills in daily work

### A different conversation

#### Surprise over the families’ approach in the conversation

The RNs were surprised by the new aspects of the family members, and their openness during the conversations. The families’ stories were rich, and they seemed to have a great need to discuss things with the RNs, which amazed them. Even in those families in which the RNs did not anticipate problems, issues surfaced that were totally new to the RNs.

If we hadn't conducted the conversations, we would think everything was just fine.

#### Silence as a tool

The RNs described that previously, in their daily work, they typically asked questions but did not always wait for the answers, because they believed they already knew the answer or had the solution to a certain problem. The RNs explained that they learned to be quiet and listen to what was actually said. They also said that being silent and just listening to the families’ stories facilitated the families to narrate things that were new to the RNs.

Well, that was just that you have to learn to be quiet and listen and not drivel on.

#### Focus on the family

The RNs described how the conversations—as focused on all family members, not just the resident—differed from the conversations they were used to having. All family members had the chance to speak and to listen to each other's situations and points of view, revealing families’ expectations and needs as they mutually influenced each other's situations.

So, I think they steered the conversations or they talked about things that they experienced as important—that's the thing; the families should be allowed to bring up what they perceive as important.

### Conversations as a creator of relationship

#### Improved understanding of the families

The RNs emphasized that they have come to know the families in a new way, which they had not previously taken the time to do. This improved their relationships with the families. The RNs also found it easier to narrate problematic and emotional issues with the families after the FamHC. In addition, it had become easier to relate to the families. The RNs explained that the FamHC series had changed their view of the family—they came to see the family as a unit, which altered their understanding of it.

They became a family, and when you combine this with a view of the resident as he was before, you gain an understanding of many things.

#### Family as a resource

Prior to the FamHC series, the RNs described that family members of a resident could sometimes be seen as troublesome. After conducting the FamHCs, the RNs described that they were instead more apt to view the families as a resource. The RNs’ changed views and beliefs of the families may imply to a new approach involving families in the care of their ill family member.

Now I see the significant others as a resource; previously I saw them as something difficult.

### To grow in the role of nurse

#### Improved self-confidence when conducting the FamHC

The RNs noted that they feel they have developed professionally by having the opportunity to use the FamHC concept in their job with the families. The RNs felt empowered over their skill improvement. They described moving from a sense of uncertainty to a feeling of comfort and confidence after having conducted all of the sessions in the conversation series.

I was strengthened by managing this; I felt more confident.

#### Developing communication skills

The RNs said that their communication skills had improved from the first sessions to the last sessions in the conversation series. At the beginning, the RNs experienced a fear of not posing questions correctly, particularly reflection and open questions, fearing they would instead become caught up in details or pose leading questions. However, at the end of the conversation series, they felt more confident in their ability to conduct the conversations.

I was a bit worried in the beginning about whether I could do it.

#### Being mentored

The RNs described being unaccustomed to leading this kind of conversation but stated that their main objectives were to carry out the conversations as competently as possible. The support of a colleague with greater experience imbued the RNs with a feeling of security when conducting the conversations. The RNs expressed a great need to have an experienced colleague with whom to share ideas and speak between the conversations.

It was great to have an experienced colleague who sat next to me and who could support me; we talked a lot before and after each conversation and reflected on the conversations.

#### Apply their skills in daily work

After a while, the RNs described the conversations as a natural part of their work. The RNs noted that after receiving the education and conducting the conversations, they used their new knowledge on other occasions, for example, when a new person moved into the residential home. They began posing more reflective questions than before and using their knowledge in other job-related conversations in their daily work.

I have found myself using what I have learned several times.

## Discussion

In this study, our aim was to describe how RNs experienced to participate in and conduct the intervention FamHCs with families in residential homes for older people. The main findings from both the diary notes and the interviews point in the same direction, that is, the RNs felt content with family conversations as a professional tool for them to use in their work with families. Within this intervention, the RNs experienced that the family members and the RNs reciprocally influenced each other in a system that entails an improved relationship with each other. When conducting the FamHC series, the RNs experienced a process of skill improvement. During the conversations, the RNs learned to listen without interrupting, which they felt was difficult and stressful when they first began conducting the conversations. In the beginning, they felt nervous and uncertain because it was a different way to converse. After conducting some FamHCs, however, it became easier for the RNs to let the families narrate and listen to the thoughts and feelings they expressed. The RNs experienced changed communication patterns both with and within the families. This conforms to other studies, many of which have noted that the RNs’ new method of communication with families had an influence on their interactions and the communication with families (Leahey, Harper-Jaques, Stout, & Levac, 1995; LeGrow & Rossen, 2005).

The RNs realized the power of silence, which gave both the RNs and the families’ time to process, as well as room for reflections and new thoughts to grow. Listening to what the families are really saying is an important skill to develop (cf. Andersen, 1995; Benzein et al., 2008; Meiers & Tomlinson, 2003). Our findings are in line with Buber's (1993) ideas about what a conversation is. He believes that just talking and not listening is not a conversation. In a genuine conversation, he states, one sometimes do not need to say a single word, and it will still be a conversation. In our findings, the RNs also emphasized non-verbal communication, with attentive listening and the slow tempo of the conversations. A confirming communication is raised through both non-verbal and verbal communication (Sundin & Jansson, 2003). The RNs expressed that when open and reflective questions were used, a number of different tales were told by the family members. The RNs realized that reflective questions open up new thoughts and make the family members aware of their own and the other family members’ beliefs. This is in line with Andersen (1995), who states that reflective questions should depart from the families’ own beliefs and reflections and that should relate to cognitive, affective, behavioral issues, and/or future issues. This is also in line with Ricæur's (1992) philosophy that, by reflecting and narrating, one reflects upon and becomes aware of one's own beliefs. Narratives in and of themselves can have a great impact on the healing process.

Reflecting during the conversations helped the RNs in our study to better understand their own and the families’ beliefs. Wright and Bell (2009) describe the importance of understanding one's own beliefs, often unconscious and unreflective experiences, in being able to understand those of others. Own beliefs that are constraining can then be explored, challenged, and altered to create a context for change. Our findings showed that the RNs’ beliefs were challenged by conducting the FamHCs.

The RNs found that within the FamHCs, they and the families developed a dialogue. Previously, the RNs’ professional conversations with the families were more like a monologue. This finding is in line with Bubers’ (1995) theory that a dialogue can only arise from a genuine encounter, when being present in, and acutely aware of, each other and each other's presence. As the RNs learned by conducting FamHC, a genuine meeting turns people toward each other, with the intention to enact reciprocity and thus understand each other. The RNs learned, as Buber (1995) also describes, that an important part of a dialogue is listening to each other's views without trying to question them.

The genuine meetings between the RNs and the families in our study made it easier for them to talk with each other, which in turn changed the RNs’ views of the families. Furthermore, through attentive listening, the nurses experienced that room was created for families to tell their stories and for the RNs to really listen to what was said. The RNs expressed that it was through dialogue that a genuine meeting occurred when conducting the conversations. According to Buber (1994), the moment when a genuine dialogue emerges depends on one's view of people. A dialogue can only be created by affirming each other and seeing the other person as a subject—as a person—and not as an object. Furthermore, a genuine meeting can only occur if you meet people in a way that is non-hierarchical and mutually confirming, as the RNs experienced the FamHC. Buber (1988) notes that confirming means, first of all, accepting the whole potentiality of the other person. Through the dialogue that took place within the FamHCs, the RNs’ understanding of families has improved, and the RNs came to see conversations with the whole family as a systemic way for the families’ experiences of their situations to become an important tool in the professional work of supporting the whole family and reaching a better understanding of the residents and their family situation. This is in line with a study by St John and Flowers (2009), indicating that using this kind of non-judgmental approach to working with families helped the nurse understand and reinforced their beliefs that involving families in care is important.

### Methodological considerations

The methods for data collection and analysis can be assumed to be relevant, as the aim was to describe RNs’ experiences in conducting FamHCs with families in residential homes for older people. The twofold perspective of the findings, the initial sense of each conversation (from the diary notes) and the reflective experiences after conducting 12 conversations (from the interviews), supplemented each other in that they make the understanding of the process of the RNs’ experiences more complete. The diary notes can provide insightful spontaneous data and can therefore complement the interviews. Diaries can also help provide access to the participants’ interpretation of a situation (Alaszewski, 2006; Thomas, 2015). The diaries consisted of notes written after each conversation, a total of 36 entries, which were rich in content. The interviews gave a good variation in the RNs responses. The sample size of three RNs could have been larger. However, because this way of working with families was new for the nurses, we tested the FamHC intervention with one nurse from each unit in this first step. This was to get knowledge about the nurses’ experiences of conducting FamHC before starting the intervention on a larger scale, if this turned out well. To enhance trustworthiness, all interviews were conducted by the same person, using an interview guide (Graneheim & Lundman, 2004). All authors discussed each step together in the analysis process of both the diary notes and the interviews until consensus about the findings was reached. All authors also reflected independently upon the findings in relation to the interview texts and the diary notes to ensure that nothing had been missed. Krippendorff (2004) asserts that a text never contains only one single meaning. Our interpretation of these findings is only one of many interpretations, but our intent is that the findings in this study may be transferred to similar contexts.

## Conclusion

The findings show that the involved RNs experienced that FamHC, an FSN nursing approach, may be a functional professional tool for them in their work. This first evaluation of how RNs experience conducting FamHC at residential homes for older people shows that it may be useful in nursing care in this context. Conducting family conversations entailed a greater understanding of the family situation and improved relationships with the families. However, the findings suggest that the development of skills for this new means of conversing with residents’ families involves a learning process for RNs. This learning process may take time and money and may also require mentorship from more experienced RNs. In the long run, this new method of communication is cost effective (Lämås, Sundin, Jacobsson, Saveman, & Östlund, 2016) and gives the RNs an improved working situation. For nurses, it might be significant to expand the approach of patient-centered care to a family-centered approach in which also the whole family is viewed and focused, that is, FSN, as all family members’ reciprocal influences on each other. In this small study, we concluded that nurses found FamHCs to be a useful professional tool for them when working with families in the context of residential care for older people. However, more empirical research is needed to expand the knowledge of how to implement and work with FSN nursing interventions in general, and FamHCs in this specific context.
